# Evaluating Bacterial Pathogenesis Using a Model of Human Airway Organoids Infected with Pseudomonas aeruginosa Biofilms

**DOI:** 10.1128/spectrum.02408-22

**Published:** 2022-10-27

**Authors:** Mingxing Tang, Shumin Liao, Jing Qu, Yixin Liu, Shuhong Han, Zhao Cai, Yunping Fan, Liang Yang, Shuo Li, Liang Li

**Affiliations:** a Department of Otolaryngology, Huazhong University of Science and Technology Union Shenzhen Hospital, Shenzhen, China; b School of Medicine, Southern University of Science and Technology, Shenzhen, China; c Institute of Biomedicine and Biotechnology, Shenzhen Institutes of Advanced Technology, Chinese Academy of Sciences, Shenzhen, China; d Department of Otolaryngology, The Seventh Affiliated Hospital of Sun Yat-sen University, Shenzhen, China; e Department of Pathogen Biology, Shenzhen Center for Disease Control and Prevention, Shenzhen, China; South China Sea Institute of Oceanology

**Keywords:** human airway organoids at air-liquid interface, *Pseudomonas aeruginosa* biofilm, dual-species transcriptomics, quorum sensing, NF-κB inflammatory response

## Abstract

Pseudomonas aeruginosa is one of the leading invasive agents of human pulmonary infection, especially in patients with compromised immunity. Prior studies have used various *in vitro* models to establish P. aeruginosa infection and to analyze transcriptomic profiles of either the host or pathogen, and yet how much those works are relevant to the genuine human airway still raises doubts. In this study, we cultured and differentiated human airway organoids (HAOs) that recapitulate, to a large extent, the histological and physiological features of the native human mucociliary epithelium. HAOs were then employed as a host model to monitor P. aeruginosa biofilm development. Through dual-species transcriptome sequencing (RNA-seq) analyses, we found that quorum sensing (QS) and several associated protein secretion systems were significantly upregulated in HAO-associated bacteria. Cocultures of HAOs and QS-defective mutants further validated the role of QS in the maintenance of a robust biofilm and disruption of host tissue. Simultaneously, the expression magnitude of multiple inflammation-associated signaling pathways was higher in the QS mutant-infected HAOs, suggesting that QS promotes immune evasion at the transcriptional level. Altogether, modeling infection of HAOs by P. aeruginosa captured several crucial facets in host responses and bacterial pathogenesis, with QS being the most dominant virulence pathway showing profound effects on both bacterial biofilm and host immune responses. Our results revealed that HAOs are an optimal model for studying the interaction between the airway epithelium and bacterial pathogens.

**IMPORTANCE** Human airway organoids (HAOs) are an organotypic model of human airway mucociliary epithelium. The HAOs can closely resemble their origin organ in terms of epithelium architecture and physiological function. Accumulating studies have revealed the great values of the HAO cultures in host-pathogen interaction research. In this study, HAOs were used as a host model to grow Pseudomonas aeruginosa biofilm, which is one of the most common pathogens found in pulmonary infection cases. Dual transcriptome sequencing (RNA-seq) analyses showed that the cocultures have changed the gene expression pattern of both sides significantly and simultaneously. Bacterial quorum sensing (QS), the most upregulated pathway, contributed greatly to biofilm formation, disruption of barrier function, and subversion of host immune responses. Our study therefore provides a global insight into the transcriptomic responses of both P. aeruginosa and human airway epithelium.

## INTRODUCTION

Pseudomonas aeruginosa is a common biofilm-forming opportunistic pathogen that causes pulmonary infections in immunocompromised individuals, particularly for those with cystic fibrosis (CF) (1). If untreated, P. aeruginosa pulmonary infection can lead to high rates of morbidity and mortality. Such clinical situations often involve biofilm growth that confers bacteria with strong resistance against the antibiotics and host immune responses, which makes eliminating P. aeruginosa infection a grave challenge (2, 3).

Once infections are established, P. aeruginosa can produce multiple virulence factors that severely impair airway epithelium tissue and subvert host immune responses (4, 5). These include tight junction (TJ) destruction by elastase LasB (6) and rhamnolipids (7), endoplasmic reticulum stress by pyocyanin (8), protein synthesis inhibition and apoptosis/necrosis by exotoxin ToxA (9), respiratory dysfunction by hydrogen cyanide (10), disruption of the actin cytoskeleton by ExoS (11, 12), and interference of phagocytosis by ExoT (11). Bacterial virulence depends largely on the protein secretion system for translocating those virulent effectors to target host cells (13). The expression of those toxins is under the fine-tuning control of complex regulatory networks, including quorum sensing (QS), two-component systems (TCSs), and the secondary messenger cyclic-di-GMP. The pathogenicity and regulation of these virulence factors have been well studied using model animals (14) or monococultures of tumor-like cells (15); however, to what extent these experimental conditions are relevant to human remains to be clarified. On the other hand, a few *in vivo* transcriptomic studies based on clinical samples have captured bacterial transcriptomic profiles during chronic infection, but extensive variations are observed (16, 17). Thus, a highly humanized airway tract is of great importance to model P. aeruginosa infection and study the bacterial pathogenesis.

Primary human nasal, tracheal, and bronchial epithelial cells, obtained from biopsies or surgery, can be subsequently differentiated at the air-liquid interface (ALI) and form an *ex vivo* organotypic model. When induced and cultured properly, these cells self-organize to constitute a pseudostratified epithelium layer, including many distinct functional cell types (e.g., basal, ciliated, and mucus-secreting goblet cells) (18–20). These cultures closely recapitulate architectural, physiological, and transcriptomic features of the native human airway epithelium (21–23). Although some early studies have cocultured those ALI cultures and P. aeruginosa to unravel the pathogenic impact of the type III secretion system (24), flagellin (25), and rhamnolipids (7), it warrants further investigation to determine the critical virulence pathways that are important in bacterial biofilm formation, injury of airway epithelium, and modulation of the host immune responses.

In this study, we cultured and differentiated primary human airway organoids (HAOs) that were derived from healthy donors. Using these cultures, we established an acute P. aeruginosa infection model and carried out dual-species transcriptome sequencing (RNA-seq) analyses. The gene expression profiles of the HAOs highlighted the activation of multiple inflammatory signaling, while those of P. aeruginosa revealed QS as the most upregulated genetic circuit. We further analyzed the impacts of QS on bacterial biofilm formation, tissue disruption, and immune evasion. We conclude that HAOs are a highly reliable and relevant model of the native human respiratory tract and that P. aeruginosa QS dominates during the host-pathogen interaction.

## RESULTS

### HAOs were used as a model system to monitor P. aeruginosa infection dynamics.

**FIG 1:**
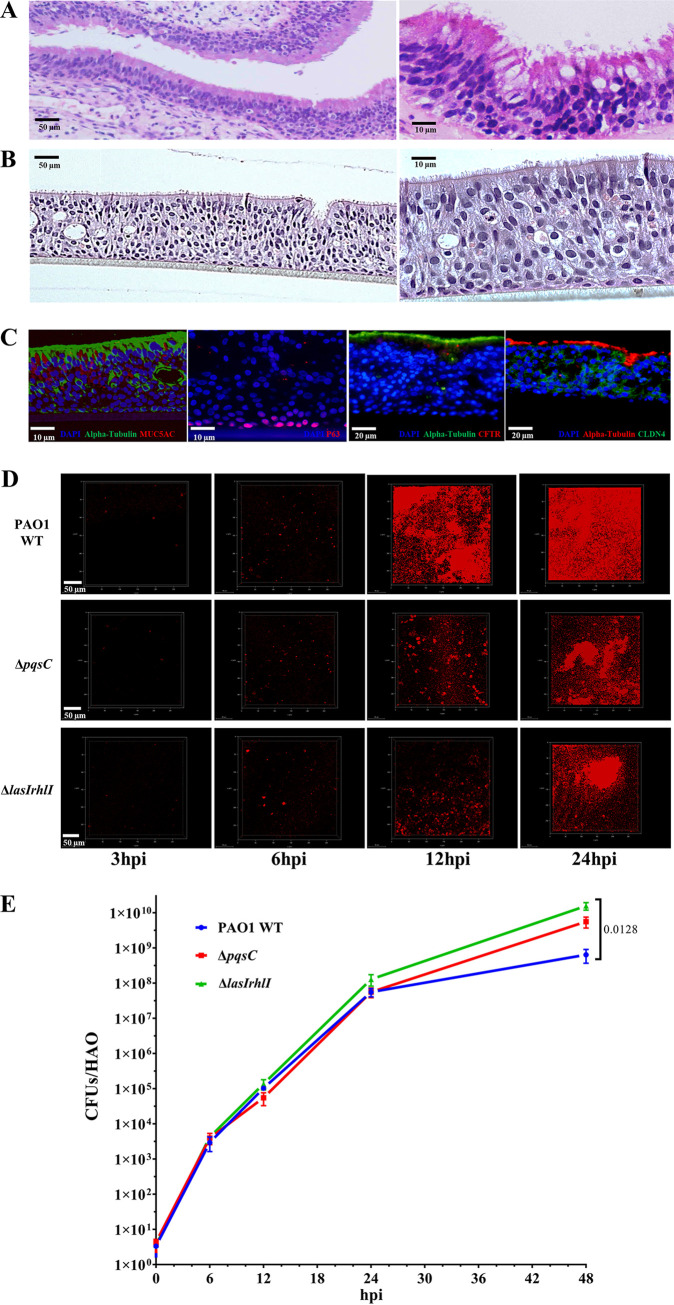
Modeling P. aeruginosa infection on HAOs. Histology of healthy human airway epithelium (A) and HAOs at the air-liquid interface (ALI) stained with H&E (B). (C) Immunofluorescent (IF) detection of acetyl-α-tubulin, MUC5AC, p63α, CFTR, and CLDN4 in the HAOs, with nuclei stained using DAPI. Scale bars = 10, 20, or 50 μm. (D) P. aeruginosa expansion imaged using fluorescence microscopy at 3, 6, 12, and 24 hpi. Red, P. aeruginosa strains labeled with a *lac*::mCherry plasmidic fusion. Scale bar = 50 μm. (E) Bacterial growth curve was measured as the number of CFU recovered from HAOs. Data are shown as mean ± SEM from three independent experiments done in triplicate. *P* values were calculated by one-way analysis of variance (ANOVA) with Tukey’s posttest for multiple comparisons.

In initial experiments, we followed the standard protocols described in Materials and Methods and cultured six different batches of HAOs (designated N15, N27, N28, N59, N60, and N63), which originated from the primary airway tract epithelial cells of six human donors. To characterize the cellular morphology and differentiation of HAOs, we prepared tangential sections of native airway epithelium (Fig. 1a) and HAO cultures (Fig. 1b), which were stained with hematoxylin-eosin (H&E). The apical cells from both types of epithelia were of high resemblance, mostly columnar in shape, and featured multiple cilia (Fig. 1). Immunofluorescence (IF) staining of these cells confirmed the expression of α-tubulin, a marker that is essential for cilium development (Fig. 1c). Intermittently arranged with the multiciliated cells, goblet cells were identified by the IF of MUC5AC staining (Fig. 1c), which is involved in mucus production. In contrast, the cells at the basolateral layers were smaller and more compact. These cells were negative for both α-tubulin and MUC5AC staining but positive for p63 (Fig. 1c), an important marker for epithelium tissue development (26). Additionally, Claudin 3, one representative tight junction protein, was detected at the intercellular space, indicative of well-preserved physical barrier (Fig. 1c). To summarize, our HAO cultures were highly polarized and structurally resembled the native human airway epithelium.

Next, we attempted to model a dynamic infection process of HAOs by the P. aeruginosa laboratory strain PAO1 and to monitor, in real-time, the colonization and biofilm development. Expansion of bacteria that had been added onto the apical surface was then observed using confocal laser scan microscopy at different time points within a 24-h period. In line with previous findings (7, 27), P. aeruginosa biofilm formation initiated as early as 3 h postinoculation (hpi), with a few bacterial microcolonies present on the HAOs despite a very low initial inoculum amount of 3.8 CFU on average (Fig. 1). PAO1 proliferated rapidly and scattered preferentially at the intercellular spaces between multiciliated cells of the HAOs (Fig. 1d). Stable bacterial expansion was observed as the infection time progressed to 6 and 12 hpi (Fig. 1d). The bacteria developed a greater number of microcolonies, while some of them expanded and connected to adjacent ones, together developing into larger clusters (Fig. 1d). At 24 hpi, a confluent mature biofilm was observed (Fig. 1d). To further validate the observed bacterial expansion, bacteria from the apical surface of the HAOs at 3, 6, 12, 24, and 48 hpi were recovered and plated to quantify the CFU; and the results supported a stable replication of PAO1 on the HAOs (Fig. 1e).

### Dual RNA-seq analyses of HAOs and P. aeruginosa following 24 h of coculturing.

**FIG 2:**
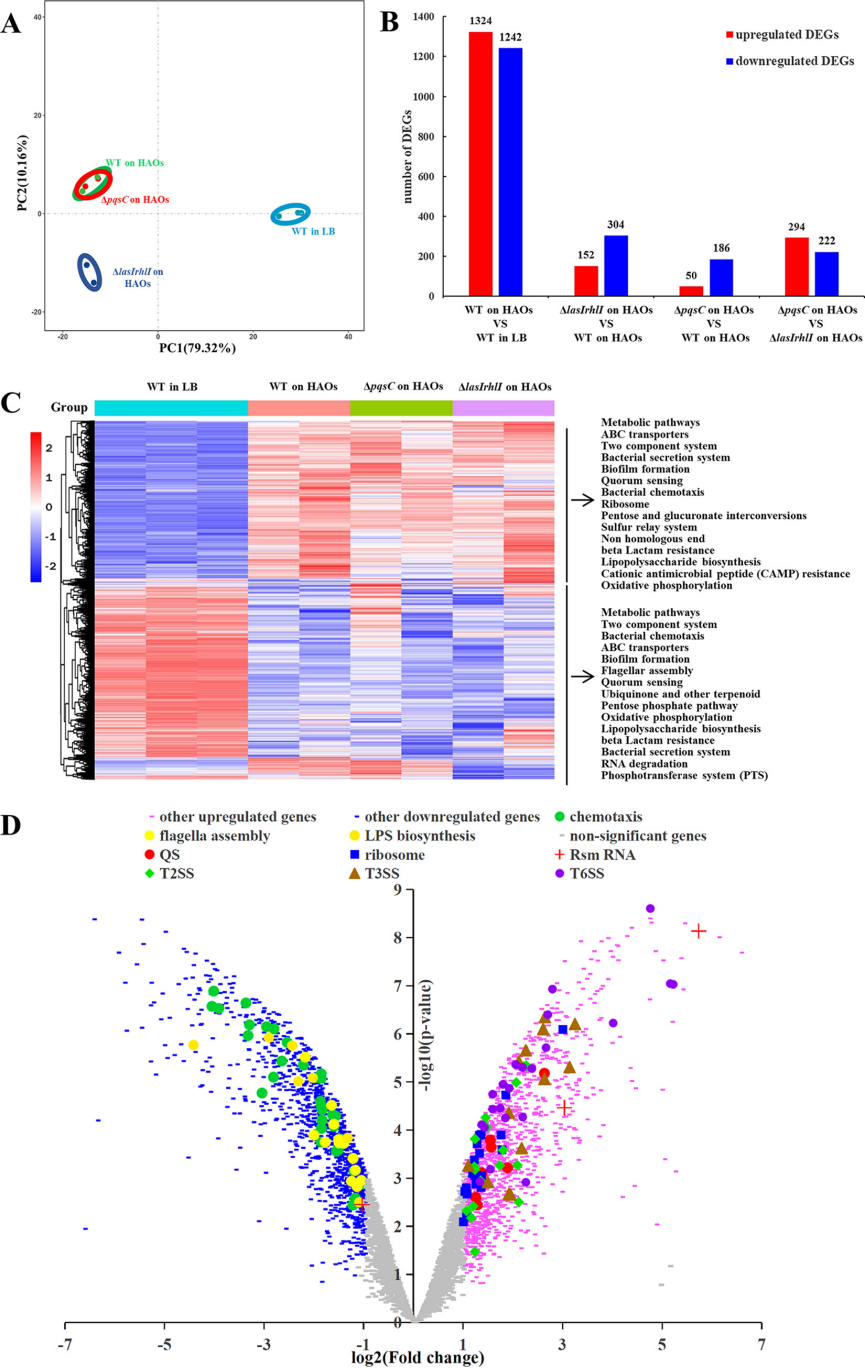
Global transcriptomic analysis of P. aeruginosa on the HAOs at 24 hpi. (A) PCA plot for differentially expressed genes in all of the P. aeruginosa samples. (B) Number of genes differentially expressed in the different paired groups. Red columns, numbers of upregulated genes; blue columns, numbers of downregulated genes. (C) Heat map of differentially expressed genes, normalized for sequencing depth across all samples. The top 30 enriched KEGG pathways are listed on the left. (D) Volcano plot of differentially expressed genes in PAO1 WT grown on HAOs, relative to PAO1 in LB medium.

We next investigated how gene expression changed on both sides at this host-pathogen interface by collecting the coculture of P. aeruginosa and HAOs to perform a dual-species transcriptional analysis at 24 hpi, a time point at which mature biofilm formation by PAO1 was observed. We compared the transcriptional profile of PAO1 wild type (WT) on HAOs to that on an abiotic surface to select the infection-specific genes. Principal-component analysis (PCA) plotting revealed close concordance between the samples within the same group, but marked divergence appeared when the HAO-associated PAO1 was compared with its counterpart grown in Luria Bertani (LB) broth (Fig. 2a). PAO1 on HAOs exhibited an extensive transcriptional change for pathogenesis, with 2,566 genes differentially expressed (DEGs; 1,324 upregulated, 1,242 downregulated) (Fig. 2b). As suggested by the QS reporter gene expression assays (see Fig. S1a in the supplemental material), QS in the WT bacteria on HAOs was operative at 24 hpi; interestingly, the expression magnitude of a few QS regulators (*lasI* and *rhlR*) was significantly higher than those grown in LB (Fig. 2c). Other well-known master regulators for virulence, biofilm formation, and metabolite transportation were also upregulated in the WT bacteria on HAOs, including two small RNAs (*rsmY* and *rsmZ*), alginate biosynthesis regulator *algD*, and an ensemble of TCSs (Fig. 2d and Fig. S1b). Of special interest, the HAO-associated WT bacteria upregulated a substantial number of genes involved in type II, type III, and type VI secretion systems (T2SS, T3SS, and T6SS, respectively) (Fig. 2d and Fig. S1b), which are responsible for translocating an arsenal of cytotoxic effectors into either extracellular space or target cells upon cellular contact (13, 28). Additionally, genes related to the ribosome (*rpl* and *rps* clusters) were also upregulated significantly (Fig. 2d and Fig. S1b), suggesting profound bacterial replication at 24 hpi that was in accordance with the bacterial growth at this time point (Fig. 1e). In contrast, genes relevant to flagellar assembly, bacterial chemotaxis, lipopolysaccharide (LPS) biosynthesis, and a group of TCSs were significantly downregulated in PAO1 grown on HAOs (Fig. 2d and Fig. S1c). Altogether, these transcriptional changes correlated with a bacterial lifestyle transition from motility to sessility, biofilm development, and HAO-associated pathogenesis.

**FIG 3:**
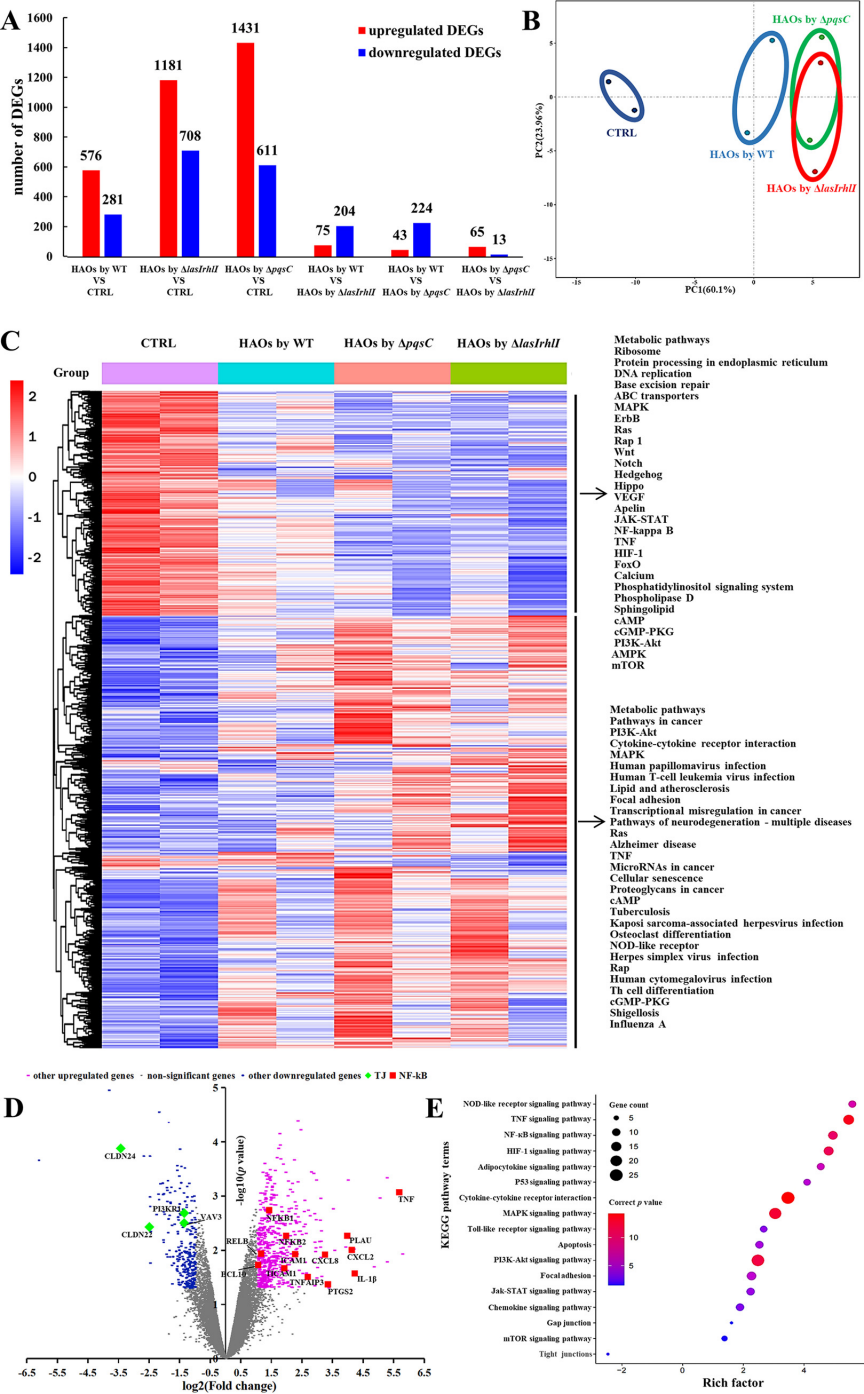
Global transcriptomic analysis of HAOs that were exposed to P. aeruginosa at 24 hpi. (A) Number of genes that were differentially expressed in different paired groups. Red columns, numbers of upregulated genes; blue columns, numbers of downregulated genes. (B) PCA plot for differentially expressed genes in all the HAO samples. (C) Heat map of differentially expressed genes, normalized for sequencing depth across all samples. The top 60 enriched KEGG pathways are listed on the left. (D) Differential expression analysis of WT exposed and unexposed HAO cells. Differentially expressed genes (adjusted *P* < 0.01) are highlighted in blue (log_2_[fold change] ≤ −1, downregulated) and pink (log_2_[fold change] ≥ 1, upregulated); genes relevant to regulation of TJs and the actin cytoskeleton and the NF-κB signaling pathway are specifically labeled. (E) Several inflammation/apoptosis-associated immune responses were upregulated in the PAO1 WT-infected cells, relative to the unexposed cells. Normalized enrichment score (NES) for gene sets significantly enriched (adjusted *P* value) in each comparison are shown, with positive values denoting upregulation and negative values representing downregulation.

To define how the HAOs simultaneously responded to P. aeruginosa infection, we compared the expression profiles between the cells infected with PAO1 and those that had never been exposed to bacteria. Analyses from both the number of DEGs (Fig. 3a) and PCA plotting (Fig. 3b) revealed that the HAOs that had been subjected to PAO1 infection were remarkably divergent from their counterparts that were devoid of infection, illustrating that PAO1 infection massively altered the host gene expression. Similar to previous findings (25), the NF-κB signaling pathway lay at the center of the immune response of the airway epithelium to P. aeruginosa infection (Fig. 3c). A considerable number of genes encoding inflammatory cytokines and chemokines, including interleukin-6 (IL-6), IL-1α, CXCL3, and CCL20, were overrepresented (Fig. 3d). In addition to NF-κB, genes upregulated in the infected group were also enriched in many other inflammatory pathways, including tumor necrosis factor alpha (TNF-α) signaling, mitogen-activated protein kinase (MAPK), phosphatidylinositol 3-Akt (PI3-Akt), mTOR, hypoxia, and cytokine-cytokine receptor interaction and NOD-like receptor signaling (Fig. 3e); most of them are either essential for or coupled with NF-κB activation in airway epithelium upon P. aeruginosa infection (29–32). Moreover, a few genes related to cellular cytoskeleton rearrangement and TJ were significantly downregulated, suggesting barrier disruption of the HAOs at the transcriptional level (Fig. 3e).

### QS systems had a pronounced effect on biofilm morphology.

Based on the transcriptomic profile of P. aeruginosa PAO1 on HAOs, we attempted to assess the impact of QS on the bacterial infection behavior, including expansion and biofilm formation. To this end, we first constructed a series of isogenic QS mutants of PAO1 and monitored the transcription dynamics of three QS reporter genes (*lasB*, *rhlA*, and *pqsA*). The *in vitro* bacterial growth of all tested strains was similar (see Fig. S2a in the supplemental material), while the expression of reporter genes decreased stepwise in the WT, Δ*pqsC*, and Δ*lasIrhlI* (Fig. S2b). Therefore, the mutants, Δ*pqsC* and Δ*lasIrhlI*, were selected to infect the HAOs for subsequent investigations. To determine whether QS would affect bacterial proliferation on the HAOs, we recovered bacteria from the apical surface of the HAOs for CFU enumeration. The QS mutants exhibited stable bacterial expansion kinetics similar to that of the WT at the tested time points (Fig. 1d and e), indicating that QS had little effect on bacterial growth on HAOs within 48 h. Like the WT, these two QS mutants also developed microcolonies at 3 and 6 hpi, as observed using confocal laser scan microscopy (Fig. 1d). They were, however, defective in migrating and progressively developing into a mature biofilm at 12 hpi and thereafter (Fig. 1d).

**FIG 4:**
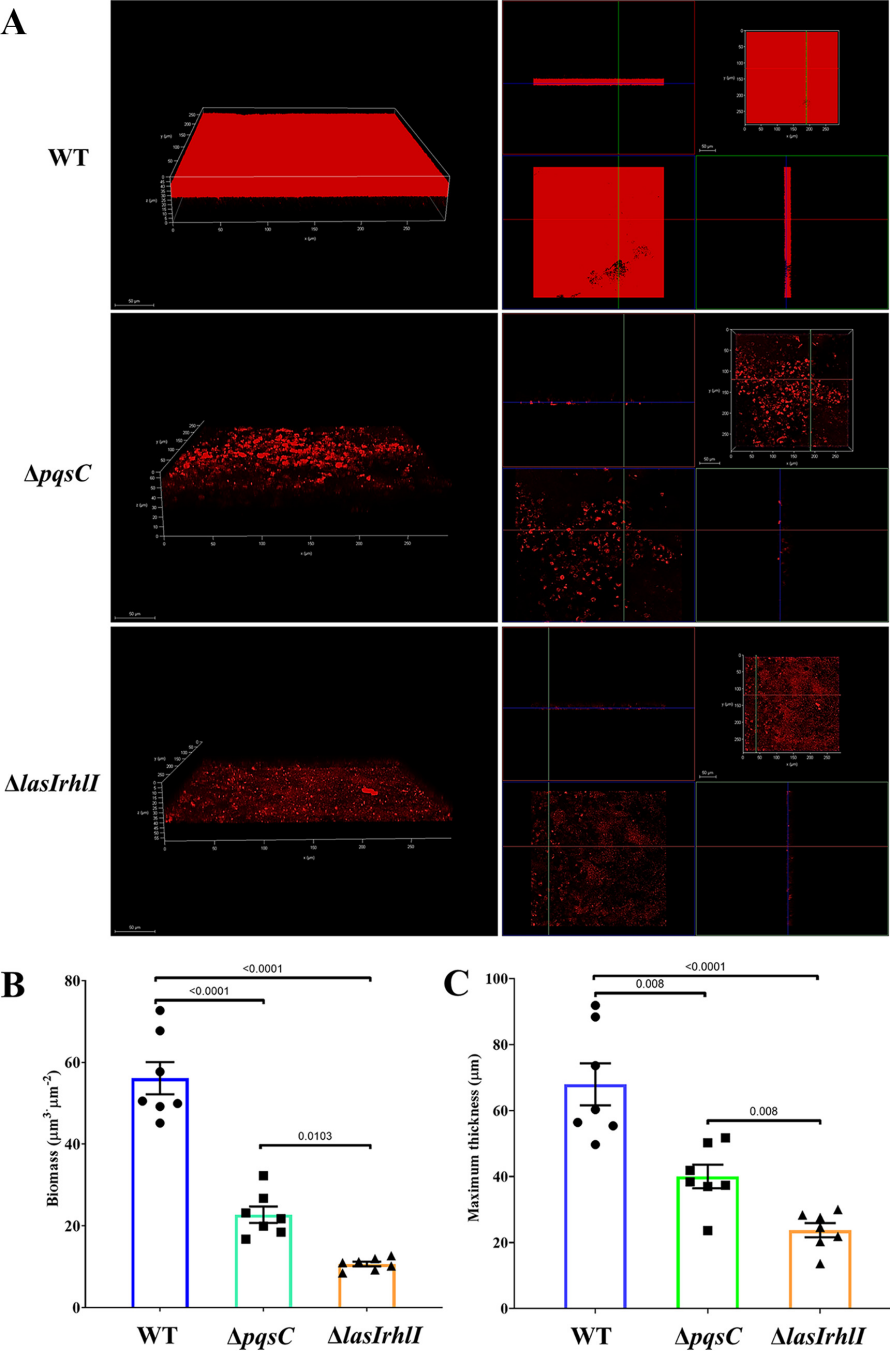
P. aeruginosa biofilm grown on HAOs at 24 hpi. (A) (Left) Volumetric projection of representative images of P. aeruginosa biofilm. (Right) Projections of XZ (blue-green crossline), XY (red-green crossline), and YZ (blue-red crossline) of the corresponding images. Scale bar = 50 μm. The biomass (B) and maximum height (C) of the P. aeruginosa biofilm grown on HAOs at 24 hpi were quantified using the Comstat program. Data are shown as mean ± SEM from three independent experiments. *P* values were calculated by one-way ANOVA with Tukey’s posttest for multiple comparisons.

To better resolve the biofilm morphology, we performed z-stacked imaging of the biofilms grown on the apical surface of HAOs at 24 hpi. The WT developed a dense and thick biofilm (Fig. 4a), with an average biomass of 55 μm^3^·μm^−2^ (Fig. 4b) and a maximum height of 78.5 μm (Fig. 4c). In contrast, Δ*pqsC* and Δ*lasIrhlI* formed only scattered microcolonies, rather than mature biofilms (Fig. 4a), with profoundly lower biomass and maximum thickness (Fig. 4b and c). Ample evidence has revealed that QS can affect P. aeruginosa biofilm formation at the *in vitro* surface, but this regulatory effect is tightly dependent on the conditional nutrients (33). To clarify this impact in our model, we rinsed the apical face of the HAOs with sterile phosphate-buffered solution (PBS) to obtain the lavage liquid, which was then used to culture PAO1 strains to monitor their growth. Consistent with the CFU enumeration data, the growth curves of the QS-defective mutants greatly resembled that of the WT bacteria grown in the lavage liquid of the HAO apical surface (see Fig. S3a in the supplemental material). WT and the mutants differed significantly, however, in biofilm biomass following 24 h of growth (Fig. S3b). To summarize, QS contributed to PAO1biofilm formation in the apical microenvironment of the HAOs.

### QS systems enabled P. aeruginosa to damage the cellular architecture of HAOs but did not affect the intracellular uptake by host cells.

**FIG 5:**
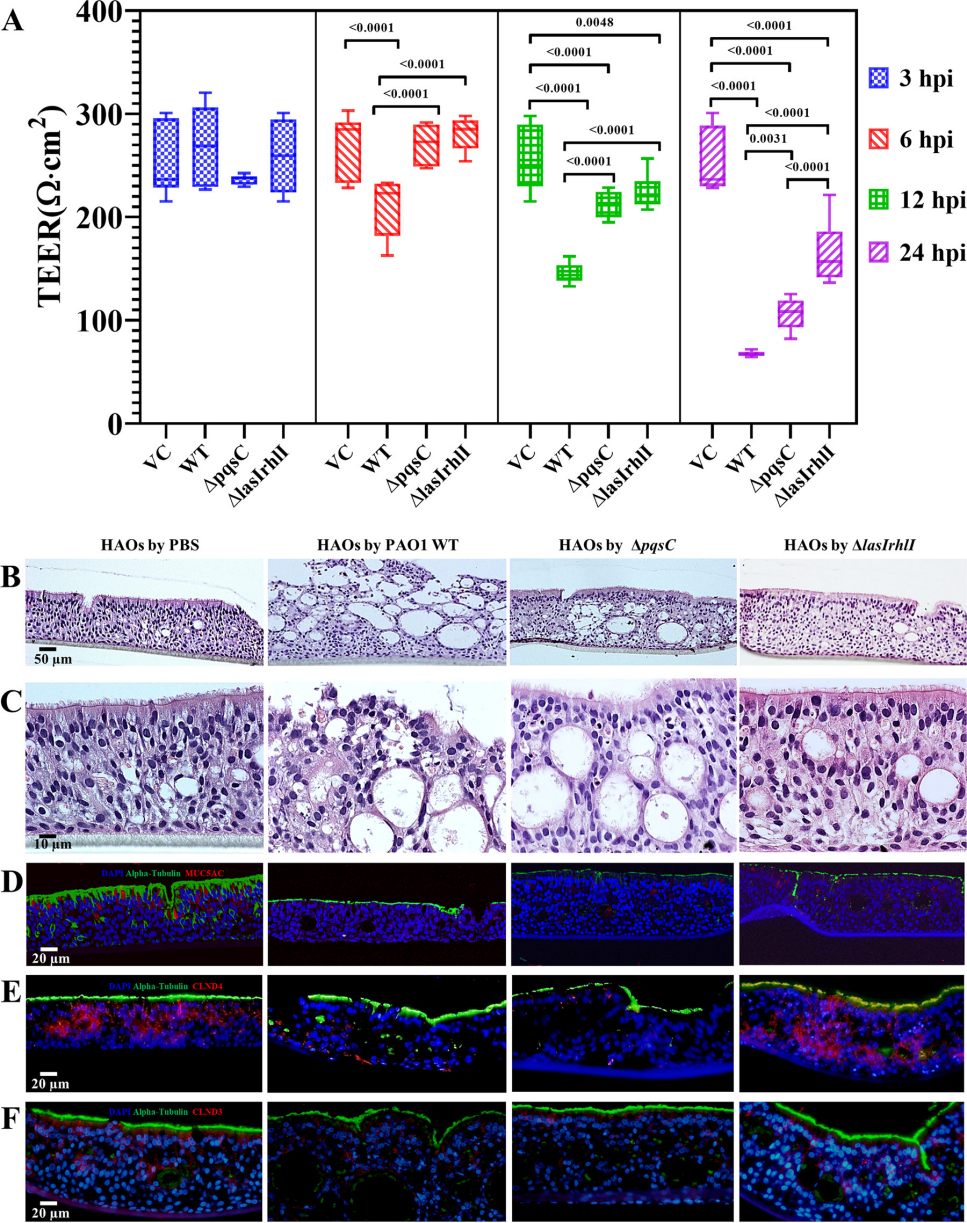
Cellular architecture of HAOs disrupted by P. aeruginosa infection. (A) TEER significantly decreased over time after exposure of the HAO apical surface to the P. aeruginosa strain PAO1 or its isogenic QS mutants. Values were presented as means ± SEM; *P* values were calculated by one-way ANOVA with Tukey’s posttest, compared with the PAO1 WT strain. (B and C) H&E-stained histopathology of tangential sections of the HAOs that had been subjected to treatment with PBS or invasion of P. aeruginosa PAO1 for 24 h. HAO sections were stained using immunofluorescence with antibodies of acetyl-α-tubulin and MUC5AC (D), Claudin 4 (E), and Claudin 3 (F). Nuclei were counterstained using DAPI. Sizes of scale bars are indicated in the images.

Dual RNA-seq analyses indicated a highly active operation of QS in PAO1 and disruption of the junction barrier in HAOs. To quantify the barrier disruption of HAOs upon PAO1 infection, the transepithelial electrical resistance (TEER) value was monitored along with the infection process. Compared with the control, the TEER in the PAO1 WT-infected group remained unchanged at 3 hpi but decreased rapidly from 6 hpi and gradually reached a low value of approximately 67.75 Ω·cm^2^ at 24 hpi (Fig. 5a). Nevertheless, when the HAOs were exposed to the Δ*pqsC* and Δ*lasIrhlI* mutants, the reduction in the TEER was postponed to 12 hpi and the drop was less dramatic than that of the WT group (Fig. 5a). These results indicated a profound role of QS in the devastation of the airway epithelium permeability.

To better resolve the HAO histopathology caused by PAO1 infection and to verify the role of QS in tissue damage, we stained (H&E) tangential sections of the HAOs infected with either the WT or QS mutants at 24 hpi. The cellular architecture of the HAOs devoid of bacterial infection remained intact (Fig. 5b and c). In striking contrast, the PAO1 WT-infected HAOs were irregularly arranged and severely disrupted, with evident collapse in the apical cell layers (Fig. 5b and c). Immunostaining with anti-α-tubulin and anti-MUC5AC also revealed dramatic losses of cilia and mucin production (Fig. 5d). Consistently, decreased immunostaining of two TJ markers, namely, Claudin 4 (Fig. 5e) and Claudin 3 (Fig. 5f), confirmed the barrier dysfunction. Similarly, infection by the Δ*pqsC* mutant also caused remarkable losses of cilia and TJs (Fig. 5). In contrast, the epithelium lesion was massively attenuated in the Δ*lasIrhlI-*infected group (Fig. 5).

A previous study reported that intracellular infection of P. aeruginosa was hardly visualized in such HAO cultures (7). This report contradicts observations from other work based on animal models (34) and tumor cell lines (15, 35, 36). To clarify this point in our HAO cultures and to examine whether QS affected bacterial endocytosis, we labeled bacteria in the HAO cells using P. aeruginosa-specific fluorescent *in situ* hybridization (FISH) probes, while staining the host cell cytoskeleton with an anti-β-actin antibody. Confocal laser scan microscopy clearly revealed that the a few bacteria were localized in proximity of the nucleus and surrounded by the cytoskeleton (see Fig. S4 in the supplemental material), which is indicative of intracellular P. aeruginosa reservoirs. The intracellular infection was also observed in the Δ*pqsC-* and Δ*lasIrhlI*-infected HAOs (Fig. S4), indicating that QS deficiency did not abrogate the bacterial internalization. Furthermore, we also detected the expression of the cystic fibrosis transmembrane conductance regulator (CFTR), an ion channel protein that has been postulated to mediate P. aeruginosa internalization (37–39) (Fig. 1c). To summarize, our HAO model of P. aeruginosa infection recapitulated crucial events of such a human-pathogen interaction and underscored the important roles of QS in disrupting the barrier function.

### P. aeruginosa QS deficiency reshaped gene expression profiles of both the host and pathogen.

**FIG 6:**
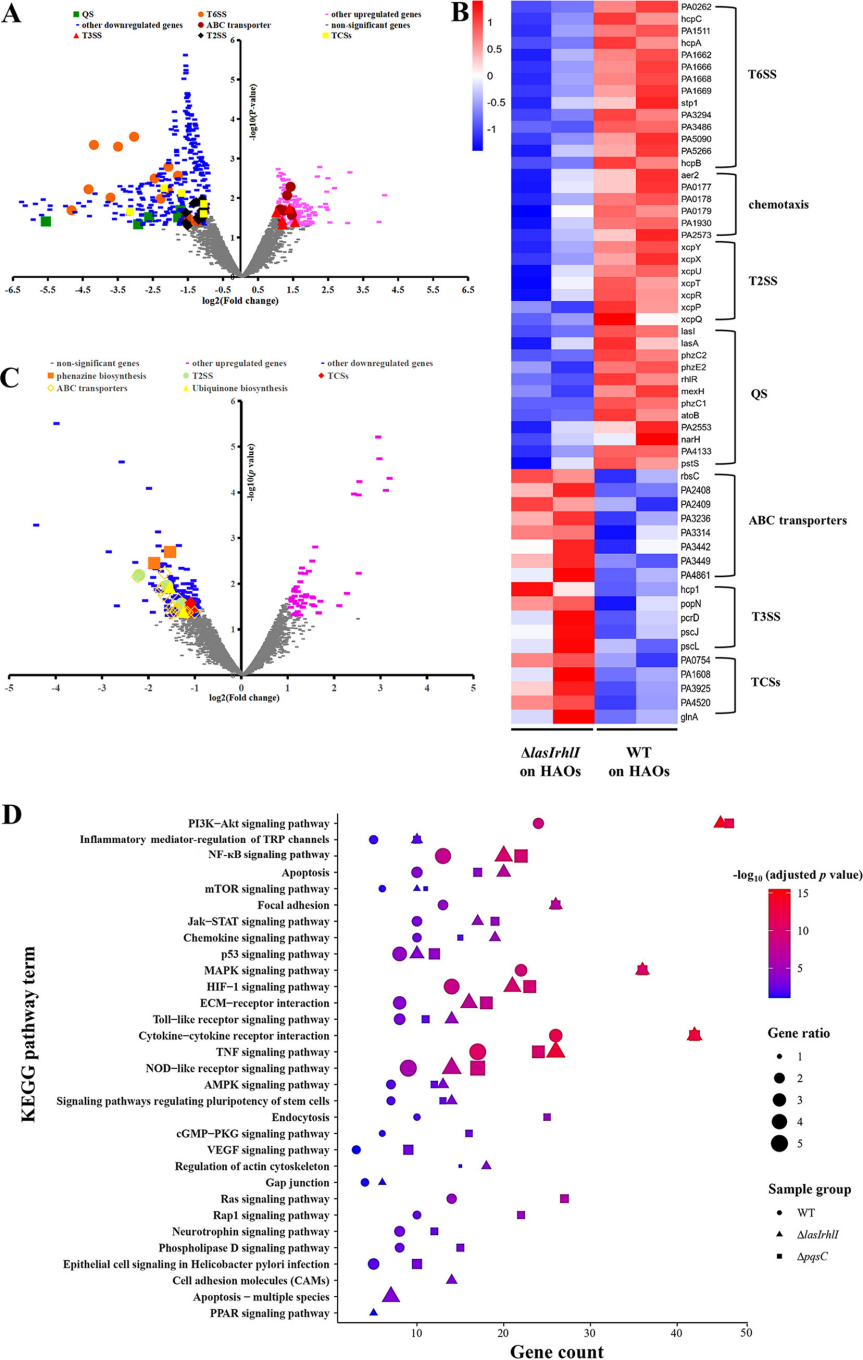
Quorum sensing depletion reshaped the gene expression of both PAO1 and HAOs. (A) Differentially expressed genes (Padj < 0.01) are highlighted in blue (log_2_[fold change] ≤ −1, downregulated) and pink (log_2_[fold change] ≥ 1, upregulated) in the Δ*lasIrhlI* mutant relative to WT bacteria following 24 h of infection; genes relevant to T2SS, T3SS, T6SS, ABC transporters, QS, and TCSs are specifically labeled. (B) Specific gene components and functional categories that were upregulated and downregulated in Δ*lasIrhlI*, relative to PAO1 WT. (C) Differentially expressed genes (Padj < 0.01) are highlighted in blue (log_2_[fold change] ≤ −1, downregulated) and pink (log_2_[fold change] ≥ 1, upregulated) in Δ*pqsC* relative to WT bacteria. (D) Enrichment of selected KEGG pathways based on significantly upregulated genes in HAO samples infected by different bacterial strains, relative to the vehicle control (VC) group. Solid circles, PAO1 WT-infected group; solid triangles, Δ*lasIrhlI*-infected group; solid squares, Δ*pqsC*-infected group. The *x* axis indicates the number of upregulated genes mapped in each pathway. Values of −log_10_ (normalized *P* value) from low to high are indicated by colors from blue to red. Gene ratio is indicated by dot size.

Because QS affects the capacity of PAO1 to form a mature biofilm and to disintegrate the cellular architecture of HAOs, we investigated how QS depletion could reshape the transcriptional responses of both the pathogen and host. Compared with the extensive transcriptomic shift of PAO1 WT from the *in vitro* conditions to the infection state, fewer DEGs were found between the WT and Δ*lasIrhlI* mutant when both were grown on the HAOs (Fig. 2b). Within expectation, the most downregulated genes in the Δ*lasIrhlI* mutant were involved in QS, such as regulators (*lasI* and *rhlR*), phenazine biosynthesis, T2SS, and T6SS (Fig. 6a). In contrast, genes upregulated in Δ*lasIrhlI* were associated mostly with T3SS, ABC transporters, and the metabolism of various nutrient substrates (Fig. 6a and b). The expression and translocation of T3SS effectors might be the major virulent determinant of this mutant causing tissue damage, as indicated by the histopathological observation and the decrease in the TEER (Fig. 5a). Furthermore, the gene expression profile of the Δ*pqsC* mutant was strikingly similar to that of the WT (Fig. 2a). Only a few genes related to TCSs, T2SS, and the biosynthesis of ubiquinone and phenazine were significantly downregulated (Fig. 6c), with all being known regulons of the Pqs QS system (40, 41).

On the host side, bacterial QS depletion failed to cause a remarkable alteration in transcriptomic profiles of HAOs. The biological replicas of the HAO cultures clustered well by donor rather than by the association with various PAO1 strains (Fig. 3b). This finding was in line with a previous report that the highly differentiated HAO cultures retained transcriptomic signatures of human origins (42). Infection of HAOs by either the WT or the QS mutants similarly activated an array of inflammatory signaling pathways (Fig. 6d). Notably, the magnitudes of those pathways in the WT-infected group were significantly lower. Upregulated genes mapped in the inflammation-associated pathways in the QS mutant-infected HAOs significantly outnumbered those in the WT-infected ones (Fig. 6d). To validate this result, we performed reverse transcriptase quantitative PCR (RT-qPCR) assays to assess the RNA transcript levels of genes associated with the NF-κB pathway. Expression of those inflammatory mediators was significantly higher in the QS mutant-infected groups (see Fig. S5 in the supplemental material). Given the similar bacterial loads of the WT and QS mutants on the HAOs at 24 hpi (Fig. 1e), PAO1 QS operation probably lessened the inflammatory response, suggesting that QS contributes to bacterial immune evasion at the airway epithelium.

## DISCUSSION

In recent years, cocultures of human airway epithelium and P. aeruginosa have been applied to study either bacterial behavior or host responses at the host-pathogen interface (7, 24, 25, 27, 43–47). Nevertheless, it remains uncertain whether these ALI cultures can really recapitulate the crucial physiological features and provide a relevant human airway epithelial microenvironment for P. aeruginosa infection. Some ALI cultures derived from tumor-like cells (24, 27, 46, 47) or animal cells (43) have formed only a monolayer of epithelial cells, in which mucus production, cilium morphogenesis, well-organized cellular architectures, and renewal of basal cells have been completely absent (27, 43). In addition, others have been submerged in liquid culture, thereby restricting the accessibility of oxygen to both the bacteria and the host cells (44, 45). To a large extent, our HAO cultures that were derived from the primary airway epithelial cells of healthy human donors overcame these drawbacks and better simulated the native physiological traits. Apical exposure to atmosphere induced cellular polarization with cilia and mucus production, while basal cells at the basolateral side retained their capacity to regenerate.

Cell polarity is indispensable for the structural integrity and physiological function of airway epithelia. On the one hand, the primary physiological role of ciliated epithelium cells was initially thought to be essential for the transport of mucus, mucus-encased particles, and microorganisms out of the airway (48). So, it remains uncertain whether the operative mucociliary escalator could intervene the stable attachment and biofilm formation of P. aeruginosa. On the other hand, apical ferric ions released from the airway epithelium were shown to promote P. aeruginosa biofilm formation (27, 44). We therefore checked the biofilm dynamics in our HAOs, of which the apical surface was semidry, oxygen accessible, and mucus and iron rich. Consistent with previous observations (44, 45), our findings revealed that P. aeruginosa can rapidly develop a confluent and thick biofilm, whereas QS deficiency impaired the bacterial capacity to form such a mature biofilm. Indeed, several QS-controlled products, including Pel polysaccharides, rhamnolipids, extracellular DNA, and pyocyanin, are indispensable constituents of the biofilm matrix (49). Upon cellular contact with the HAOs, our study showed that PAO1 shifted its lifestyle from motility to sessility by downregulating flagella and chemotaxis, while upregulating alginate biosynthesis, with all being prerequisites for biofilm formation (4, 50–52). It would be interesting to resolve, in the future, how P. aeruginosa senses the cellular cues from the airway epithelium and in turn orchestrates gene regulation for biofilm development.

Similar to tumor cell-like cultures or animal models for P. aeruginosa infection (53), the histological investigation of P. aeruginosa-infected HAOs in this study highlighted the critical role of QS in undermining the cellular architecture and disrupting the barrier function, probably by controlling the secretion of an arsenal of cytotoxic effectors. Our transcriptomic analysis for the HAO-associated P. aeruginosa also revealed a significant upregulation of QS-controlled T2SS and T6SS. T2SS is responsible mainly for the release of virulence factors, including exotoxin A, LasA, LasB, type IV protease, phospholipase H, and lipolytic enzymes (13, 54, 55). Among them, the role of LasB elastase in disrupting TJs has been well demonstrated (6, 56). While T6SS mediates the delivery of toxic effectors in a cell contact-dependent manner, it remains poorly understood which T6SS effector is crucial in disrupting the airway epithelium and how it works. The transcriptional analysis of HAO-associated P. aeruginosa coupled with the HAO histopathological investigation might help identify the eukaryote-specific T6SS effectors. Interestingly, T3SS was also upregulated upon bacterial contact with the HAOs, although its transcription is negatively regulated by QS (57–59). This result indicates that other regulatory circuits are likely involved in T3SS activation during the infection process.

Prior works show that the transcriptomic profile of the HAOs reflects the key molecular events for airway development and physiology of the native airway epithelium (20, 42). These findings enabled the HAOs to serve as a highly reliable and relevant model of the human airway epithelium. Consequently, modeling HAO infection by P. aeruginosa could not only uncover the innate immunity against this pathogen but also unravel the impact of QS on reshaping host gene expression. Consistent with previous observations (15, 25), the most prevalent pathway induced by P. aeruginosa infection is the NF-κB-mediated inflammatory response, likely downstream from Toll-like receptors (TLRs) after recognition of pathogen-associated molecular patterns (PAMPs) (25, 60). Intriguingly, our transcriptomic analysis coupled with RT-qPCR validation revealed that P. aeruginosa QS operation might suppress the NF-κB signaling. QS signal molecules are very likely involved in the host immunomodulation (61–63). 2-Heptyl-3,4-dihydroxyquinoline (PQS), a signal ligand activating the bacterial transcriptional regulator PqsR, can downregulate NF-κB-dependent immune responses in mouse macrophages, monocytes, and airway epithelial cells, probably by inhibiting the binding of NF-κB to its target DNA or interfering the degradation of the κB inhibitor (IκB) (64). Meanwhile, *N*-(3-oxododecanoyl)-l-homoserine lactone (3O-C_12_-HSL), another QS ligand to LasR, also modulates host immune responses, but its effect differs in various studies (65–68). Furthermore, prior studies that are based on rodent models of P. aeruginosa infection have also drawn inconsistent conclusions. The QS systems are reported to have variable effects on the infection-induced immune response, including the pulmonary bacterial burden, inflammatory cytokine expression, and immune cell infiltration (69–73). The variation might be ascribed to discrepancy in the rodent species, animal age, and inoculation method. Compared with these mono-cell cultures or animal models, our HAOs that are highly relevant to the human airway epithelium helped us to clarify the confusion and validate that deletion of either *pqsC* or *lasIrhlI* in P. aeruginosa can exacerbate the inflammatory responses. Our finding therefore correlates to some animal models of P. aeruginosa pneumonia (70, 73), explaining why QS mutants can induce more infiltration of neutrophils or macrophages to the infection site.

The NF-κB inflammatory pathway is essential for eradicating extracellular bacteria (37); intriguingly, it is also necessary for bacterial intracellular persistence within epithelial cells (15). From this perspective, our findings could explain the high frequency of loss-of-function mutations in the QS system that was often observed in clinical P. aeruginosa isolates causing chronic pulmonary infections (74, 75). The evolved bacteria might dampen their QS to turn less virulent so that tissue damage, bacterial translocation across the epithelium, and massive efflux of phagocytes would be attenuated; meanwhile, NF-κB signaling is still retained or even increased, thus selecting and favoring intracellular P. aeruginosa colonizers, which may contribute to the chronicity and recurrence of pulmonary infection.

## MATERIALS AND METHODS

### Primary human airway organoids (HAOs) at an air-liquid interface (ALI).

Biopsy samples of human nasal epithelium were obtained from individuals of different ages that had been admitted for turbinate hypertrophy or nasal polyp, in the Otolaryngology Department, Huazhong University of Science and Technology Union Shenzhen Hospital. The patients’ informed consent was obtained, and this study was approved by the ethical committee of Huazhong University of Science and Technology Union Shenzhen Hospital with the approval number IRB72656.

The HAOs were cultured as described previously (76, 77). Briefly, the biopsy samples were rinsed with precooled Dulbecco’s phosphate-buffered saline (DPBS; pH 7.4) (Thermo Fisher Scientific, USA) and disassociated overnight at 4°C in Dispase I (10 mg · mL^−1^; Sigma-Aldrich, USA) supplied with penicillin (100 U), streptomycin (0.1 mg · mL^−1^), and fluconazole (50 μg · mL^−1^). The digested cells were collected by centrifugation (5,000 rpm, 5 min) and treated with 0.25% trypsin-EDTA (2 mL; Thermo Fisher Scientific) at 37°C for 15 min. The trypsin was neutralized with Dulbecco’s modified Eagle’s medium (DMEM) (1 mL; Thermo Fisher Scientific), resuspended with PneumaCult-ex Plus medium (2 mL; Stemcell Technologies, Canada) and seeded with murine osteoblast cells (MC-3T3) in six-well plates. Following 5 days of incubation under 37°C and 5% CO_2_ air-humidified conditions, the bottom-attached cells were dissociated using Accutase solution (100 μL; Stemcell Technologies) and transferred onto collagen-coated semipermeable polycarbonate membrane inserts (0.33 cm^2^, 0.4-μm pore size; Corning, USA) that were submerged in PneumaCult-ex medium. After 4 days of growth, PneumaCult-ALI (Stemcell Technologies) was used for cell growth and differentiation. Differentiation of the air-liquid organoids in the following 2 weeks was identified through immunofluorescence (IF) with specific antibodies to acetyl-α-tubulin and MUC5AC (both from Cell Signaling Technology). Upon differentiation, the apical surface of HAOs was washed gently with sterile DPBS (200 μL) for 3 min. The eluate solution was collected and homogenized. Next, 100-μL aliquots of the eluted solution were used as the culture medium for P. aeruginosa strains to grow biofilm for 24 h in 96-well plates (Corning).

### Bacterial growth conditions and mutant construction.

The bacterial strains and plasmids used in this study are listed in Table S1 in the supplemental material. P. aeruginosa strains were grown at 37°C in Luria Bertani (LB; Sangon Biotech, PRC) liquid medium. The growth rate of the PAO1 strains in the indicated liquid medium was measured by monitoring the optical density at 600 nm (OD_600_) in 96-well plates, using a Spark multiwell reader (Tecan, Switzerland), at 37°C with shaking. Antibiotics were used at the following concentrations: 6 μg · mL^−1^ chloramphenicol, 200 μg · mL^−1^ carbenicillin, 100 μg · mL^−1^ ampicillin, and 60 μg · mL^−1^ gentamicin.

In-frame deletion of QS-related genes in PAO1 was performed using the suicide pK18 plasmid, as described previously (78). Briefly, the upstream and downstream DNA fragments of target genes were amplified by Q5 high-fidelity PCR (New England BioLabs [NEB], USA) with two pairs of primers, as detailed in Table S2 in the supplemental material. The two PCR products were purified using a HiPure PCR pure minikit (Magen, PRC) and ligated to HindIII (NEB)-digested and EcoRI (NEB)-digested suicide vector PK18 by Gibson assembly master mix (NEB) to yield resultant plasmids. After sequence confirmation, the recombinant suicide plasmid PK18 was mobilized from Escherichia coli Top10 (donor strain) to P. aeruginosa PAO1 (receptor strain) by conjugal mating with the help of the pRK600 vector and selection for gentamicin-resistant first-time homologous recombinants. Colonies were then streaked onto LB agar plates with 20% (wt/vol) sucrose (Sigma-Aldrich, USA) to select second-time homologous recombinants. The deletion mutants were verified by PCR and Sanger sequencing.

### *In vitro* transcriptional expression assay of QS reporter genes.

Overnight cultures were adjusted to an OD_600_ of 1.0 and diluted by 1:100 into fresh agrobacterium minimal medium containing 25 μg. ml^-1^ thiamine (APExBIO, USA) (ABT) (79) supplemented with 5 g · L^−1^ glucose (Sangon Biotech, PRC) and 2 g · L^−1^ Casamino Acids (Sangon Biotech). Bacterial strains were grown in ABTCG medium with an initial inoculum of OD_600_ of 0.01. An aliquot (100 μL) was transferred to a polystyrene, black 96-well plate (Corning, USA). Arbitrary fluorescence intensity units (FIUs) were measured with an excitation wavelength of 488 nm, and the cell density (OD_600_ values) of different samples was simultaneously measured. Relative fluorescence units (RFUs) were determined by normalizing the FIU values against OD_600_ values as reported previously (78). Three biological repeats were performed, and each biological repeat contained at least four technical replicates.

### Construction of dual-fluorescence transcriptional fusions.

The *lasB* (80), *pqsA* (81), and *rhlA* (82) promoter-reporter fusions were amplified separately through Q5 high-fidelity PCR, using the primers detailed in Table S2. The PCR products were purified in a HiPure PCR pure minikit and ligated to the HindIII-digested pUCp20 by Gibson assembly master mix to yield resultant plasmids. Next, the promoter-reporter fusion of *lac*-mCherry was amplified and integrated into the EcoRI-digested plasmids. The corresponding plasmids were introduced into either the PAO1 wild-type (WT) strain or the QS mutants by electroporation. Transformants were selected on LB agar plates supplemented with carbenicillin.

### Infection of HAOs with P. aeruginosa strains and imaging biotic biofilm.

To establish the coculture of P. aeruginosa and HAOs, overnight cultures of bacterial strains in LB were reinoculated in fresh medium for 4 h of growth. Then, 500 μL of the bacterial culture was washed twice with sterile phosphate-buffered saline (PBS; Sangon Biotech, PRC), and the quantity of bacterial cells was counted by a Cytoflex S flow cytometer (Beckman Coulter, USA) (78). The apical side of HAOs was inoculated with 10 μL of bacterial culture diluted with PBS that contained approximately 5 bacterial cells.

To quantify the overall bacterial biomass associated with HAOs at indicated time points, the whole polycarbonate membrane was excised and submerged into a 500-μL sterile PBS solution, which was subject to 5 min of vigorous vortexing. Serial dilutions were plated onto LB agar plates for CFU numeration.

Biofilms were grown using the PAO1 wild-type strain and various mutants carrying a plasmid that constitutively expressed the fluorescent mCherry protein (Table S1). After 24 h of coculture, the apical surface of HAOs was gently rinsed twice with 300 μL sterile PBS to remove the free-living bacteria. The whole polycarbonate membrane supporting HAOs and the P. aeruginosa biofilm was excised and mounted on glass slides (Citoglas, PRC). The biotic biofilms were imaged on a Leica TCS SP8 microscope (Germany).

### *In vitro* assay of biofilm biomass.

Overnight cultures of P. aeruginosa PAO1 and various isogenic mutants were diluted to an OD_600_ of 0.01 in fresh LB medium. The inoculums (100 μL) were aliquoted into a 96-well microtiter plate in triplicate and incubated statically for 24 h at 37°C for biofilm formation. The biofilm was washed twice with sterile filtered water. Next, 0.1% crystal violet (CV; 150 μL; Sangon Biotech, PRC) was added to each well and then incubated for 13 min at room temperature for staining. The wells were washed twice thoroughly with sterile water. The CV stain was dissolved into 30% acetic acid (200 μL). The relative biofilm biomass was quantified by measuring the optical density of the CV staining at 550 nm, using a microplate reader (78).

### Measurement of epithelial barrier.

The transepithelial electrical resistance (TEER) of the reconstituted HAOs was assessed using a Millicell ERS-2 Volt-ohm meter (World Precision Instruments, USA). At indicated times, the TEER value was monitored after apical addition of PneumaCult-ALI medium (500 μL).

### Hematoxylin-eosin (H&E) staining.

The HAO cultures were fixed overnight in aqueous 4% paraformaldehyde (PFA; Beyotime, PRC), washed with PBS, embedded in paraffin, sectioned, and mounted on slides. H&E staining was performed following a standard protocol used in our previous studies (76, 77).

### Immunofluorescence.

Paraffin sections (5 μm) of HAOs were obtained as described above. After deparaffinization and rehydration, antigen retrieval was performed by heating with citric acid (10 mmol · L^−1^; Beyotime, PRC) in a hot water bath (98°C) for 45 min. The tissue sections were subsequently washed three times in blocking solution (Solarbio, PRC). The sections were incubated with the primary antibodies overnight at 4°C. The sections were then washed three times in PBS containing 0.05% Tween 20 (PBST; Sangon Biotech, PRC), followed by incubation with secondary antibodies for 1 h at room temperature. After three washes and counterstaining with 4,6-diamidino-2-phenylindole (DAPI; Solarbio, PRC), the samples were mounted with ProLong gold antifade mountant (Invitrogen, USA). The specimens were imaged using the DM750 fluorescence microscope (Leica, Germany). Primary antibodies were employed at a dilution of 1:200, including acetyl-α-tubulin (Lys40) (D20G3) rabbit monoclonal antibody (MAb; Cell Signaling Technology, USA), acetyl-α-tubulin (Lys40) (6-11B-1) mouse MAb (Cell Signaling Technology), p63-α (D2K8X) rabbit MAb (Cell Signaling Technology), CFTR (D6W6L) rabbit MAb (Cell Signaling Technology), Claudin-3 (D7A3O) rabbit MAb (Cell Signaling Technology), Claudin-4 rabbit MAb (Abcam, UK), and MUC5AC (E3O9I) rabbit MAb (Cell Signaling Technology).

### RNA-seq and quantitative reverse-transcription PCR (qRT-PCR).

Total RNAs were extracted from the cocultures of HAOs and P. aeruginosa using TRIzol reagent (Invitrogen, USA), following methods reported previously (83). DNA digestion was conducted, following RNA extraction by DNase I. The RNA quality was determined by examining the absorbance ratio *A*_260_/*A*_280_ using a Nanodrop OneC spectrophotometer (Thermo Fisher Scientific, USA). RNA Integrity was confirmed through 1.5% agarose gel electrophoresis. Qualified RNAs were finally quantified using Qubit3.0 with a Qubit RNA broad-range assay kit (Life Technologies, USA).

For qRT-PCR, mRNA was reversely transcribed into cDNAs by using a PrimeScript RT master mix (TaKaRa Bio, Japan). SYBR green qPCR was performed using PCR master mix (TaKaRa Bio). The primers specific for human genes that encode TNF, IL-1β, NF-κB, IL-6, IL-8, CXCL10, CCL4, CCL2, IFN-β, and 18S rRNA are listed in Table S2. The expression of cytokines was normalized to 18S rRNA by using the threshold cycle (Δ*C_T_*) method; the relative expression was calculated with the vehicle control (VC; HAOs treated with 10 μL of PBS) as a reference. The assay was conducted in triplicate; means and standard deviations were calculated for each group.

Total RNAs (2 μg) were used for the preparation of a stranded RNA sequencing library, using a KC-digital stranded mRNA library prep kit for Illumina (Wuhan Seqhealth, PRC), following the manufacturer’s instruction. The kit eliminated duplication bias in the PCR and sequencing steps by using a unique molecular identifier (UMI) of eight random bases to label the preamplified cDNA molecules (84). The library products corresponding to 200 to 500 bp were enriched, quantified, and finally sequenced using a Novaseq 6000 sequencer (Illumina, USA) with a paired-end 150 (PE150) model.

For RNA-seq data analysis, the raw sequencing data were first filtered using Trimmomatic (v. 0.36); low-quality reads were discarded, and the reads contaminated with adaptor sequences were trimmed. Clean reads were further treated with in-house scripts to eliminate duplication bias introduced during library preparation and sequencing. In brief, clean reads were first clustered according to the UMI sequences, in which reads with the same UMI sequence were grouped into the same cluster. Reads in the same cluster were compared with each other through pairwise alignment, and then the reads with a sequence identity greater than 95% were extracted to a new subcluster. After all of the subclusters had been generated, multiple sequence alignment was performed to obtain one consensus sequence for each subcluster. After these steps, any error and bias introduced by PCR amplification or sequencing would have been eliminated.

The deduplicated consensus sequences were used for standard RNA-seq analysis. They were mapped to the reference genome of P. aeruginosa PAO1 (from https://www.ncbi.nlm.nih.gov/assembly/GCF_000006765.1) and Homo sapiens GRCh38 (from https://ftp.ensembl.org/pub/release-87/fasta/homo_sapiens/dna/) using STAR software (v. 2.5.3a) with default parameters. Reads mapped to the exon regions of each gene were counted by featureCounts (Subread-1.5.1; Bioconductor), and then reads per kilobase per million (RPKM) was calculated. Genes differentially expressed between groups were identified using the edgeR package (v. 3.12.1). A *P* value cutoff of 0.05 and a fold change cutoff of 2 were used to judge the statistical significance of gene expression differences. Gene ontology (GO) analysis and Kyoto Encyclopedia of Genes and Genomes (KEGG) enrichment analysis for differentially expressed genes were both implemented using KOBAS software (v. 2.1.1), with a *P* value cutoff of 0.05 to judge statistically significant enrichment.

### Statistical analysis.

Prism software v. 8.0.2 (GraphPad, USA) was used for statistical analysis and preparation of plot graphs. Statistical significance was determined using the unpaired Student’s *t* test.

### Data availability.

The RNA-seq data of P. aeruginosa and human airway organoids that support the findings of this study have been deposited in the NCBI Sequence Read Archive (SRA) with the accession numbers PRJNA835709 and PRJNA836155, respectively. The materials that support the findings of this study are available from the corresponding author upon reasonable request.
