# Supplementary material for: Evaluating Bacterial Pathogenesis Using a Model of Human Airway Organoids Infected with Pseudomonas aeruginosa Biofilms
Source: Microbiol Spectr. 2022 Oct 27;10(6):e02408-22. doi: 10.1128/spectrum.02408-22 (PMC9769610; doi:10.1128/spectrum.02408-22)
Supplement: Supplemental file 1 — Supplemental material. Download spectrum.02408-22-s0001.pdf, PDF file, 1.2 MB [file spectrum.02408-22-s0001.pdf]

Evaluating bacterial pathogenesis using a model of human airway organoids infected with *Pseudomonas aeruginosa* biofilms

Mingxing Tang[1, 2, 3#], Shumin Liao[2, 4#], Jing Qu[5#], Yixin Liu[2], Shuhong Han[2], Zhao Cai[2], Yunping Fan[4], Liang Yang[2*], Shuo Li[1*], Liang Li[2, 3*]

1. Department of Otolaryngology, Huazhong University of Science and Technology Union Shenzhen Hospital, Shenzhen, China
2. School of Medicine, Southern University of Science and Technology, Shenzhen, China
3. Institute of Biomedicine and Biotechnology, Shenzhen Institutes of Advanced Technology, Chinese Academy of Sciences, Shenzhen, China
4. Department of Otolaryngology, The Seventh Affiliated Hospital of Sun Yat-sen University, No.628, Zhenyuan Road, Guangming District, Shenzhen
5. Department of Pathogen Biology, Shenzhen Center for Disease Control and Prevention, Shenzhen, China

*Correspondence: Liang Li lil@sustech.edu.cn ; Shuo Li Shuoli@email.szu.edu.cn; Liang Yang yangl@sustech.edu.cn
#Equal contribution

Running title: Modeling bacterial biofilm on Human-Airway-Organoids

Keywords: human airway organoids at air-liquid interface, *Pseudomonas aeruginosa* biofilm, quorum sensing, dual-species transcriptomics, NF-κB inflammatory response

Legends of supplementary figures

Fig. S1 Transcriptomic profiles of PAO1 WT at 24 hpi on HAOs had shifted toward pathogenesis and biofilm formation. (A)Transient expression of three QS reporter genes (assayed by the transcriptomic fusions *lasB::gfp*, *rhlA::gfp*, and *pqsA::gfp*) of *P. aeruginosa* PAO1 on HAOs at 24 hpi. *lac::mCherry* fusion enabled a constitutive emission of red fluorescence to indicate the bacterial cell position. Scale bar: 50 μm. Heat maps indicating the specific genes and their relevant functional categories that had been (B) upregulated and (C) downregulated in PAO1 WT grown on HAOs compared to those grown in LB medium.

Fig. S2 *P. aeruginosa* phenotypic tests in ABTGC medium. (A) Bacterial growth curve measured in terms of OD_{600} and mCherry fluorescence intensity. (B) QS reporter gene expression dynamics in *P. aeruginosa* PAO1 and its isogenic mutants Δ*lasI*, Δ*rhlI*, Δ*pqsC*, and Δ*lasIrhlI*, along with bacterial growth in ABTGC medium, as measured through p*lasB::gfp*, p*rhlA::gfp*, and p*pqsA::gfp* transcriptional fusion.

Fig. S3 *P. aeruginosa* phenotypic tests in the lavage liquids of the HAO apical surface. (A) Growth curve and (B) biofilm formation of *P. aeruginosa* PAO1 and its isogenic mutants Δ*pqsC* and Δ*lasIrhlI*, quantified through CV staining. Values correspond to means ± SEM; *P* values were calculated by one-way ANOVA with Tukey's post-test, when compared with the PAO1 WT strain. Experiments performed in triplicate were repeated at least four times.

Fig. S4 HAO cells can be intracellularly infected by *P. aeruginosa*. Cytoskeletons of host cells that had been infected with *P. aeruginosa* at 24 hpi were stained with β-actin antibodies; nuclei were stained with DAPI. Projections of XZ (blue-green crossline), XY (red-green crossline), and YZ (blue-red crossline) of the serial images are provided. Scale bar: 10 μm.

Fig. S5 RT-qPCR detection of mRNA transcript levels of genes encoding regulators, cytokines, and chemokines in the NF-kB pathway (except interferon-β). Infection of HAOs by *pqsC* or *lasIrhlI* mutants induced significantly higher levels of expression of most inflammatory

markers, relative to those infected by the WT. Experiments were performed from three different batches of HAOs infected with PAO1 strains. Values correspond to means ± SEM; *P* values were calculated by one-way ANOVA with Tukey's post-test, when compared with the WT-infected HAOs.

List of supplementary tables

Table S1. Strains and plasmids used in this study

Table S2. Primers used in this study

Supplementary Table 1. Strains and plasmids used in this study

Strain or plasmid	Relevant genotype and/or characteristics	Reference
Escherichia coli strains		
E. coli TOP10	F-, *mcrA* Δ(*mrr-hsd* RMS-*mcr BC*) *φ80lacZ* Δ*M15* Δ*lac X74 recA1 ara* Δ*139* Δ(*ara*-leu) *7697 galU galK rpsL* (Strr) *endA1 nupG*	TIANGEN
E. coli RK600	Cm^R, ColE1, oriV, RK2, mob^+, tra^+; helper plasmid in triparental matings	(1)
***Pseudomonas aeruginosa* strains**		
PAO1	*Pseudomonas aeruginosa* ATCC	(2)
PAO1 Δ*lasI*	In-frame deletion of *lasI* in PAO1	This study
PAO1 Δ*rhlI*	In-frame deletion of *rhlI* in PAO1	This study
PAO1 Δ*pqsC*	In-frame deletion of *pqsC* in PAO1	This study
PAO1 Δ*lasIrhlI*	*lasI* and *rhlI* double-mutant derived from PAO1	This study
**Plasmids**		
pK18	Small mobilizable vector, Gm^R, sucrose sensitive (sacB)	(3)
puAm::p*lac*-mCherry, p*lasB*-ASV	pUCP20 containing *lasB*-gfp (ASV) reporter fusion (2) and *lac*-mCherry reporter fusion	This study
puAm::p*lac*-mCherry, p*rhlA*-ASV	pUCP20 containing *rhlA*-gfp (ASV) reporter fusion (4) and *lac*-mCherry reporter fusion	This study
puAm::p*lac*::mCherry, p*pqsA*-ASV	pUCP20 containing *pqsA*-gfp (ASV) reporter fusion (5) and *lac*-mCherry fusion	This study

References

1. Kessler B, de Lorenzo V, Timmis KN. 1992. A general system to integrate *lacZ* fusions into the chromosomes of gram-negative eubacteria: regulation of the Pm promoter of the TOL plasmid studied with all controlling elements in monocopy. Mol Gen Genet 233:293-301.

2. Hentzer M, Riedel K, Rasmussen TB, Heydorn A, Andersen JB, Parsek MR, Rice SA, Eberl L, Molin S, Høiby N, Kjelleberg S, Givskov M. 2002. Inhibition of quorum sensing in *Pseudomonas aeruginosa* biofilm bacteria by a halogenated furanone compound. Microbiology 148:87-102.

3. Schäfer A, Tauch A, Jäger W, Kalinowski J, Thierbach G, Pühler A. 1994. Small mobilizable multi-purpose cloning vectors derived from the *Escherichia coli* plasmids pK18 and pK19: selection of defined deletions in the chromosome of *Corynebacterium glutamicum*. Gene 145:69-73.

4. Yang L, Rybtke MT, Jakobsen TH, Hentzer M, Bjarnsholt T, Givskov M, Tolker-Nielsen T. 2009. Computer-aided identification of recognized drugs as *Pseudomonas aeruginosa* quorum-sensing inhibitors. Antimicrob Agents Ch 53:2432-2443.

5. Yang L, Barken KB, Skindersoe ME, Christensen AB, Givskov M, Tolker-Nielsen T. 2007. Effects of iron on DNA release and biofilm development by *Pseudomonas aeruginosa*. Microbiology 153:1318-1328.

Supplementary Table 2. Primers used in this study

Oligo ID	Sequence	Annealing temperature/ °C	Product size	Purpose
0089 lasI 001	AGCTCGGTACCCGGGGGATCCCTCGGAAGCCAATGTGAACT	60	496 bp	amplify the upstream region of lasI for gene deletion with BamHI recognization site in the forward oligos
0090 lasI 002	ACAGGTCCCCGTCATGAAACCCGACCAATTTGTACGATCA	60		
0091 lasI 003	TGATCGTACAAATTGGTCGGGTTTCATGACGGGGACCTGT	62	492 bp	amplify the downstream region of lasI for gene deletion with HindIII recognization site
0092 lasI 004	CGACGGCCAGTGCCAAGCTTAGCTGACCCTGGAACTGGAC	62		
0093 lasI 005	CTCGGAAGCCAATGTGAACT	60	if WT 1439 bp, if deleted 863bp	verify the gene deletion of lasI
0094 lasI 006	ATCGGCAACCTTACCCATCT	60		
0095 rhlI 001	AGCTCGGTACCCGGGGGATCCGAAATCGCCATCATCCTGAG	60	318 bp	amplify the upstream region of rhlI for gene deletion with BamHI recognization site in the forward oligos
0096 rhlI 002	CGAAACGGCTGACGACCTCATTCGATCATGACCAAGTCCC	60		
0097 rhlI 003	GGGACTTGGTCATGATCGAATGAGGTCGTCAGCCGTTTCG	66	487 bp	amplify the downstream region of rhlI for gene deletion with HindIII recognization site in the reverse oligos
0098 rhlI 004	CGACGGCCAGTGCCAAGCTTGCGCTCCAGGTTGATCGAGA	66		
0099 rhlI 005	GTTGCATGATCGAGTTGCTG	60	if WT 1354 bp, if deleted 760 bp	verify the gene deletion of rhlI
0100 rhlI 006	AGCTGAAGGGCTTGTAGTCG	60		
0047 pqsC 001	AGCTCGGTACCCGGGGGATCCAGGGCTATCGCAACGTCCTG	65	549	amplify the upstream region of pqsC for gene deletion with BamHI recognization site in the forward oligos
0048 pqsC 002	CCGGCCATGACGATACGATCGTAGCTGCGAGCCGGAAGTT	65		
0049 pqsC 003	AACTTCCGGCTCGCAGCTACGATCGTATCGTCATGGCCGG	65	579	amplify the downstream region of pqsC for gene deletion with HindIII recognization site in the in the reverse oligos
0050 pqsC 004	CGACGGCCAGTGCCAAGCTTGTCCGAACAGTCCATGCGCT	65		
0023 pqsC 005	ATTGGAAGTGGACAGCGAGT	60	if WT 1863 bp, if deleted 954 bp	verify the gene deletion of pqsC
0024 pqsC 006	TATCCAGTACCGGGATGTGC	60		
0051 lasB 001	ACGACGGCCAGTGCCAAGCTTGAATTCGAGCTCGGTACCCG	60	1148 bp	amplify the lasB-ASV region for cloning into puAm plasmid
0052 lasB 002	CAAGCTCAGCTAATTAAGCTTATTAAGCTCCTGCAGCGTAG	60		
0033 rhlA 001	ACGACGGCCAGTGCCAAGCTTATTCGAGCTCGGTACCCAAT	60	1491 bp	amplify the rhlA-ASV region for cloning into puAm plasmid
0034 rhlA 002	CAAGCTCAGCTAATTAAGCTTATTAAGCTCCTGCAGCGTAG	60		
0053 pqsA 001	ACGACGGCCAGTGCCAAGCTTAGGATGGCCTTCTAGGATCA	56	1263 bp	amplify the pqsA-ASV region for cloning into puAm plasmid
0054 pqsA 002	CAAGCTCAGCTAATTAAGCTTATTAAACTGCTGCAGCGTAG	56		
0143 PA FISH 001	ACTACCAGGCAGATTCCTAGGCA	60	none	Fluorescent in situ hybridization probe labeled with 6-carboxyfluorescein (6-FAM) at the 5´-end
0153 plac 001	TATAAAAAATAGACCAGGGT AATATT ACTGGAAAGCGGGCAGTGAG	56	141bp	amplify the promoter region of the lac operon for integrating into puAm plasmid
0153 plac 001	TCCTCGCCCTTGCTCACCAT AGCTGTTTCCTGTGTGAAAT			
0155 mch 001	ATTTCACACAGGAAACAGCT ATGGTGAGCAAGGGCGAGGA	60	711bp	amplify the mCherry encoding region for integrating into puAm plasmid
0156 mch 002	CATTACCCCAGGCGTTTAAG GGCACC AATAACTGCCTTAAAAAAATTACTTGTACAGCTCGTCCA			
0161 IL-1B 001	CCACAGACCTTCCAGGAGAATG	60	131bp	amplify a fragment of il-1beta for qPCR
0162 IL-1B 002	GTGCAGTTCAGTGATCGTACAGG			
0163 nf-kb 001	GCAGCACTACTTCTTGACCACC	60	130bp	amplify a fragment of nf-κb for qPCR
0164 nf-kb 002	TCTGCTCCTGAGCATTGACGTC			
0165 il-6 001	AGACAGCCACTCACCTCTTCAG	60	132bp	amplify a fragment of il-6 for qPCR

0166 il-6 002	TTCTGCCAGTGCCTCTTTGCTG			
0167 tnf-alpha 001	CTCTTCTGCCTGCTGCACTTTG	60	135bp	amplify a fragment of tnf-alpha for qPCR
0168 tnf-apha 002	ATGGGCTACAGGCTTGTCACTC			
0169 il-8 001	GAGAGTGATTGAGAGTGGACCAC	60	112BP	amplify a fragment of il-8 for qPCR
0170 il-8 002	CACAACCCTCTGCACCCAGTTT			
0171 CXCL10 001	GGTGAGAAGAGATGTCTGAATCC	60	134bp	amplify a fragment of cxcl10 for qPCR
0172 CXCL10 002	GTCCATCCTTGGAAGCACTGCA			
0173 CCL4 001	GCTTCCTCGCAACTTTGTGGTAG	60	140bp	amplify a fragment of ccl4 for qPCR
0174 CCL4 002	GGTCATACACGTACTCCTGGAC			
0175 CCL2 001	AGAATCACCAGCAGCAAGTGTCC	60	98bp	amplify a fragment of ccl2 for qPCR
0176 CCL2 002	TCCTGAACCCACTTCTGCTTGG			
0177 IFN beta 001	CTTGGATTCCTACAAAGAAGCAGC	60	146bp	amplify a fragment of ifn beta for qPCR
0178 INF beta 002	TCCTCCTTCTGGAACTGCTGCA			
0179 18S rDNA 001	GGAGTATGGTTGCAAAGCTGA	60	129 bp	amplify a fragment of 18S rRNA for qPCR
0180 18S rDNA 002	ATCTGTCAATCCTGTCCGTGT			

Figure S1

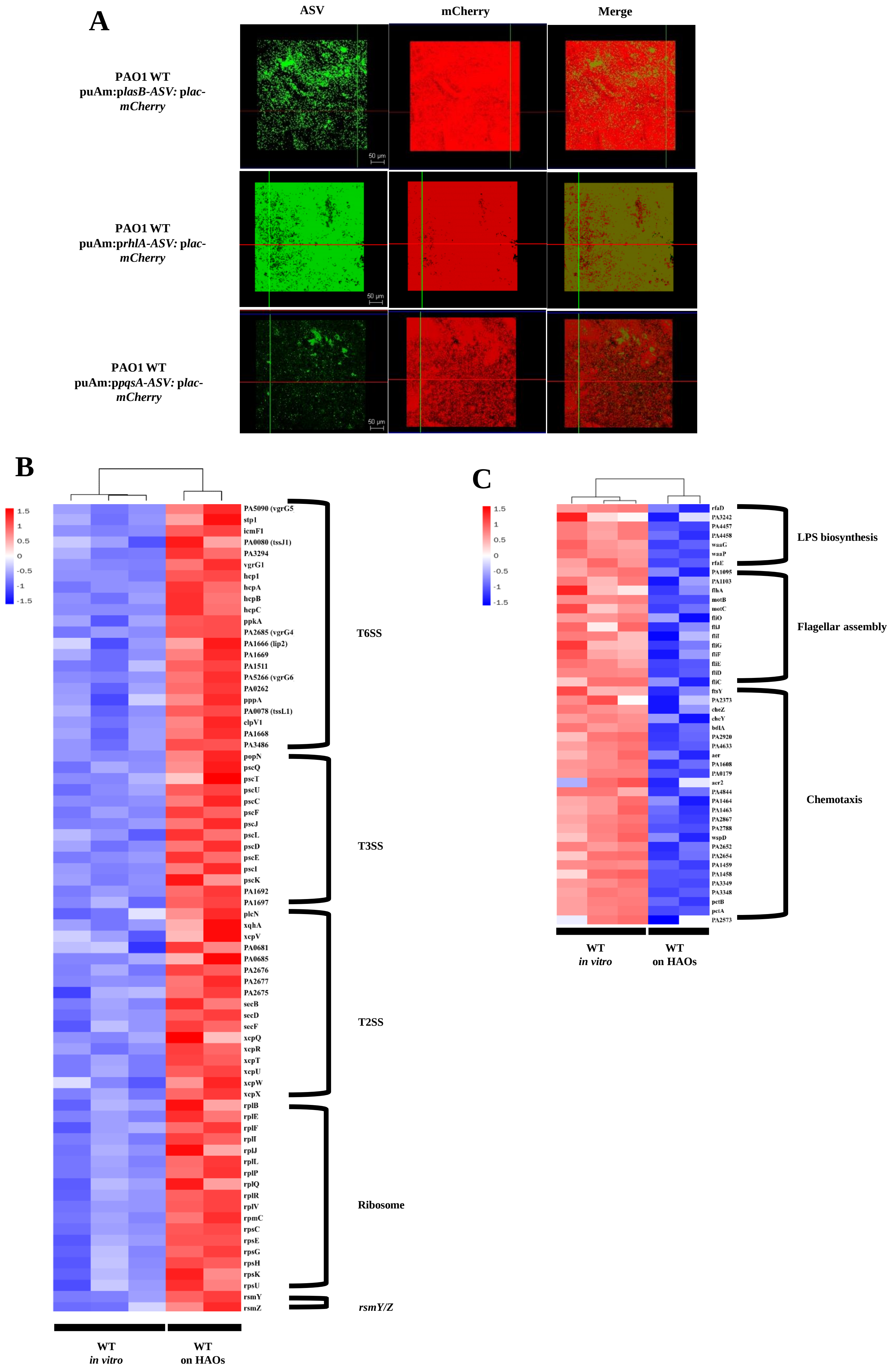
A
ASV
mCherry
Merge
PAO1 WT
puAm:plasB-ASV: plac-mCherry
PAO1 WT
puAm:prhlA-ASV: plac-mCherry
PAO1 WT
puAm:ppqsA-ASV: plac-mCherry
50 µm
B
1.5
1
0.5
0
-0.5
-1
-1.5
PA5090 (vgrG5
stp1
icmF1
PA0080 (tssJ1)
PA3294
vgrG1
hcp1
hcpA
hcpB
hcpC
ppkA
PA2685 (vgrG4
PA1666 (lip2)
PA1669
PA1511
PA5266 (vgrG6
PA0262
pppA
PA0078 (tssL1)
clpV1
PA1668
PA3486
popN
pscQ
pscT
pscU
pscC
pscF
pscJ
pscL
pscD
pscE
pscI
pscK
PA1692
PA1697
pleN
xqhA
xcpV
PA0681
PA0685
PA2676
PA2677
PA2675
secB
secD
secF
xcpQ
xcpR
xcpT
xcpU
xcpW
xcpX
rplB
rplE
rplF
rplI
rplJ
rplL
rplP
rplQ
rplR
rplV
rpmC
rpsC
rpsE
rpsG
rpsH
rpsK
rpsU
rsmY
rsmZ
T6SS
T3SS
T2SS
Ribosome
rsmY/Z
WT
in vitro
WT
on HAOs
C
1.5
1
0.5
0
-0.5
-1
-1.5
rfaD
PA3242
PA4457
PA4458
waaG
waaP
rfaE
PA1095
PA1103
flhA
motB
motC
fliO
fliJ
fliI
fliG
fliF
fliE
fliD
fliC
ftsY
PA2373
cheZ
cheY
bdlA
PA2920
PA4633
aer
PA1608
PA0179
aer2
PA4844
PA1464
PA1463
PA2867
PA2788
wspD
PA2652
PA2654
PA1459
PA1458
PA3349
PA3348
pctB
pctA
PA2573
LPS biosynthesis
Flagellar assembly
Chemotaxis
WT
in vitro
WT
on HAOs

Figure S2

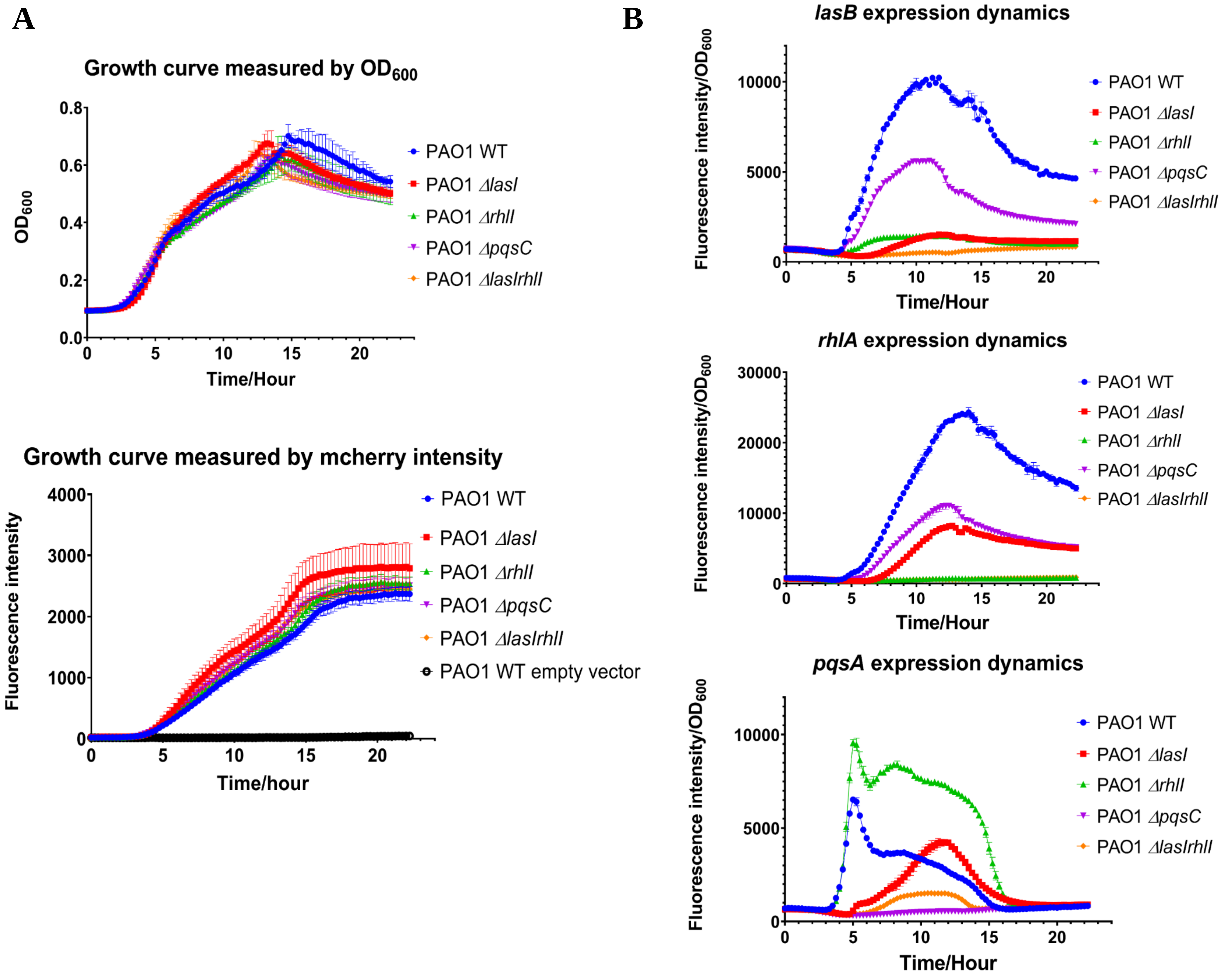
A
Growth curve measured by OD600
OD600
0.0
0.2
0.4
0.6
0.8
0
5
10
15
20
Time/Hour
PAO1 WT
PAO1 ΔlasI
PAO1 ΔrhlI
PAO1 ΔpqsC
PAO1 ΔlasIrhlI
Growth curve measured by mcherry intensity
Fluorescence intensity
0
1000
2000
3000
4000
Time/hour
PAO1 WT empty vector
B
lasB expression dynamics
Fluorescence intensity/OD600
0
5000
10000
Time/Hour
rhlA expression dynamics
20000
30000
pqsA expression dynamics

Figure S3

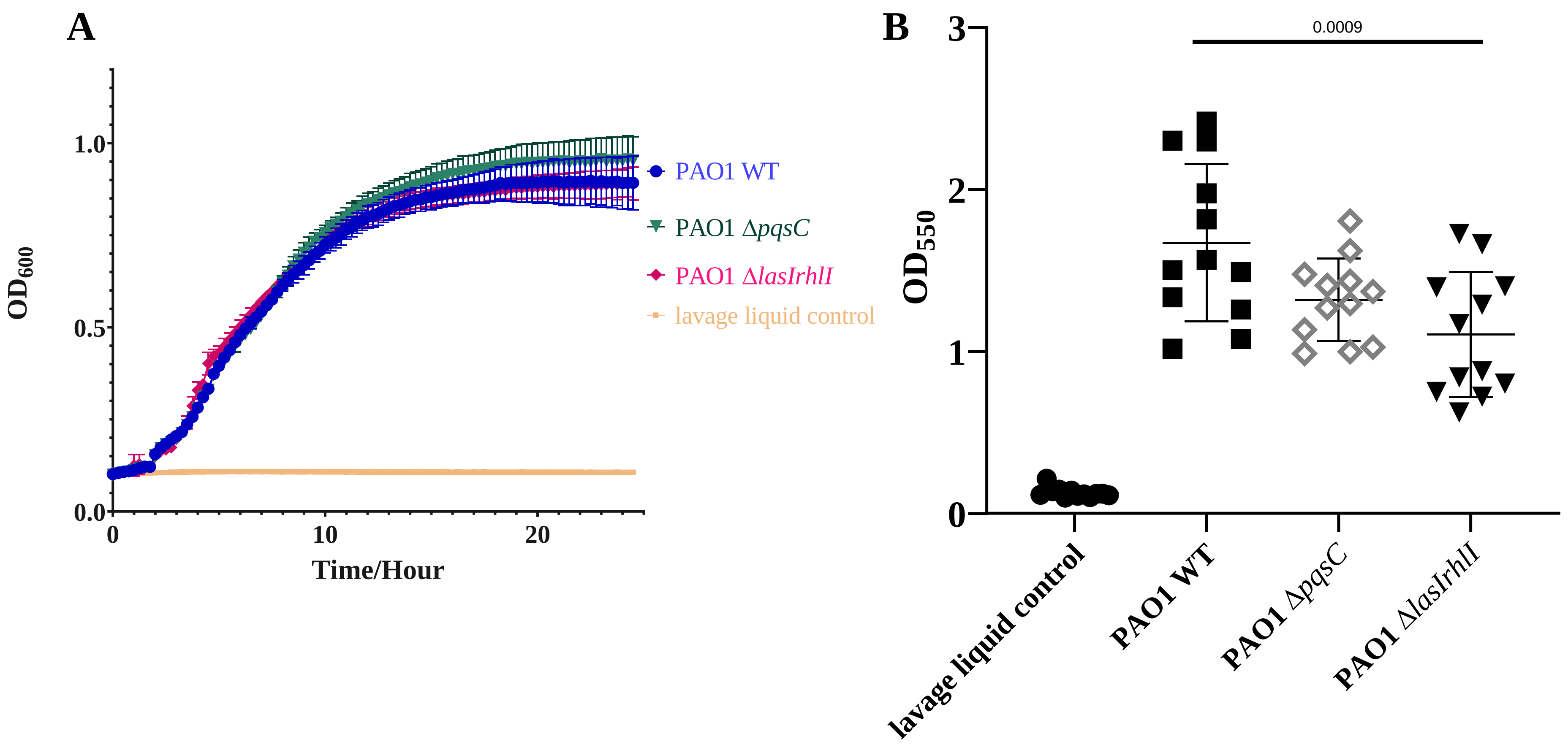

A
OD$_{600}$
1.0
0.5
0.0
0
10
20
Time/Hour
PAO1 WT
PAO1 Δ*pqsC*
PAO1 Δ*lasIrhlI*
lavage liquid control
B
OD$_{550}$
3
2
1
0
0.0009
lavage liquid control
PAO1 WT
PAO1 Δ*pqsC*
PAO1 Δ*lasIrhlI*

Figure S4

PAO1 WT-
infected HAOs

Δ*pqsC*-
infected HAOs

Δ*lasIrhlI*-
infected HAOs

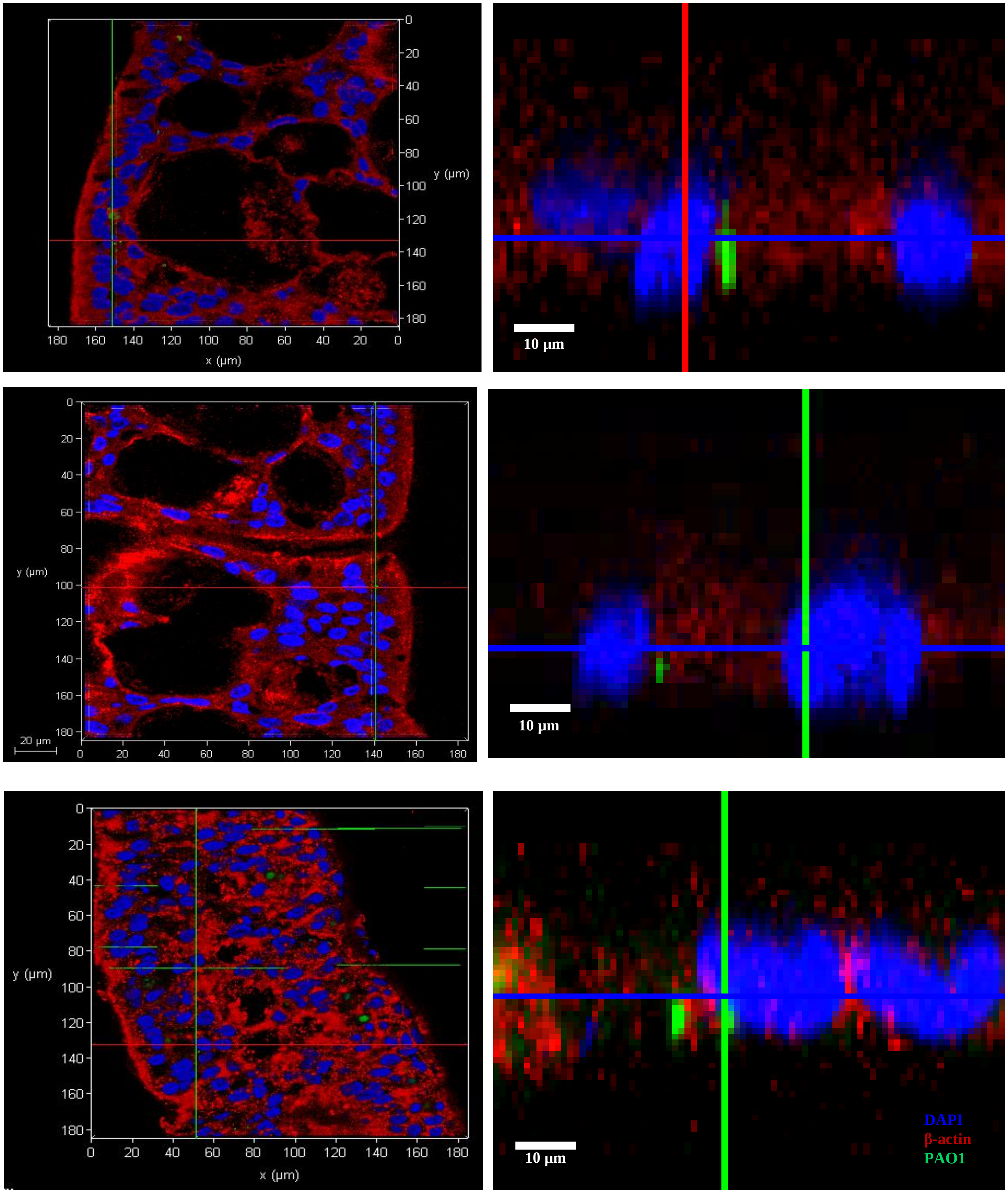

Figure S5

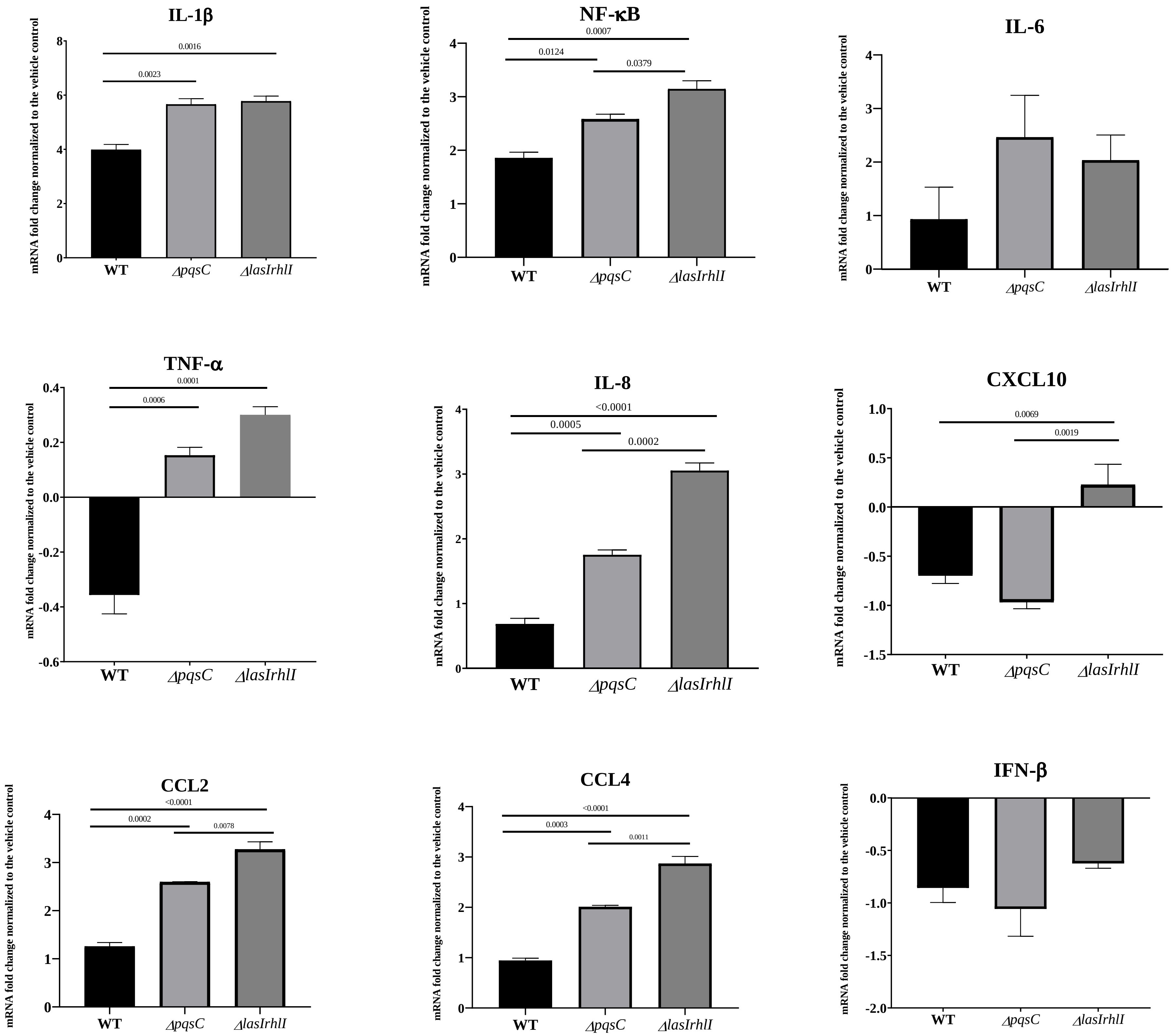
IL-1β
NF-κB
IL-6
TNF-α
IL-8
CXCL10
CCL2
CCL4
IFN-β
mRNA fold change normalized to the vehicle control
WT
ΔpqsC
ΔlasIrhlI
0.0016
0.0023
0.0007
0.0124
0.0379
0.0001
0.0006
<0.0001
0.0005
0.0002
0.0069
0.0019
0.0078
0.0003
0.0011
